# Patients risk for mortality at 90 days after proximal femur fracture – a retrospective study in a tertiary care hospital

**DOI:** 10.1186/s12877-024-04733-8

**Published:** 2024-02-03

**Authors:** Anne Postler, Charlotte Posten, Melanie Schubert, Franziska Beyer, Jörg Lützner, Oliver Vicent, Christian Kleber, Jens Goronzy, Konrad Kamin

**Affiliations:** 1 University Center of Orthopaedic, Trauma and Plastic Surgery, University Hospital, TU Dresden, Fetscherstr. 74, 01307 Dresden, Germany; 2 Department of Orthopaedic, Trauma and Plastic Surgery, University of Leipzig, Leipzig, Germany

**Keywords:** Aged, Hip Fractures, Proximal femur fractures, Surgery, Risk factors, Mortality, Survival

## Abstract

**Background:**

Despite improving the management of proximal femur fractures (PFF) with legal requirements of timing the surgery within 24 h, mortality rates in these patients remain still high. The objective of our study was to analyze potential cofactors which might influence the mortality rate within 90 days after surgery in PFF to avoid adverse events, loss of quality of life and high rates of mortality.

**Methods:**

In this retrospective, single-center study all patients with PFF aged 65 years and older were included. We recorded gender, age, type of fracture, surgery and anesthesia, time, comorbidities and medication as well as complications and mortality rate at 90 days. Separate logistic regression models were used to assess which parameters were associated with patients’ mortality. The mortality rate was neither associated with timing, time and type of surgery nor time and type of anesthesia, but with higher age (OR 1.08 per year; 95% CI 1.034–1.128), lower BMI (OR 0.915 per kg/m^2^; 95% CI 0.857–0.978), higher CCI (OR 1.170 per point; 95% CI 1.018–1.345), dementia (OR 2.805; 95% CI 1.616–4.869), non-surgical complications (OR 2.276; 95% CI 1.269–4.083) and if mobilization was impossible (OR 10.493; 95% CI 3.612–30.479).

**Results:**

We analyzed a total of 734 patients (age ≥ 65 years) who had a PFF in 2019 and 2020 and received surgery. 129 patients (17.6%) died until 90 days at an median age of 89.7 years (range 65–101 years).

**Conclusion:**

The proportion of patients who died until 90 days after surgery is still high. It is less extend influenced by surgical and anaesthesiologic factors than by patient-related factors like age or lower BMI. Physicians should be aware of the importance of avoiding adverse events and the importance of patients’ mobilization to reduce mortality and improve patients’ outcome.

## Background

The aging elderly population with longer life expectancy seems to be more active and more geriatric trauma injuries are recorded [1]. Nevertheless, trauma is the fifth leading cause of death among the elderly population and the majority of these traumas involve orthopedic injuries. Patients are therefore characterized by multiple comorbidities and frailty. Suffering from trauma injuries and especially PFF decreased mobility leads to need for long-term care [2]. The risk for subsequent osteoporotic fractures, non-surgical and surgical complications as well as mortality rate are high and mortality rates are reported about 9% after 30 days and up to 36% after one year [3, 4].

Multiple efforts are made to optimize the management in elderly patients with PFF regarding especially any modifiable treatment factors and the focus is concentrated on timing of the surgery. The Federal Joint Committee (G-BA) founded by the German government ordered surgical treatment for PPF to be performed within 24 h after hospital admittance as well as interdisciplinary treatment in order to avoid complications and reduce mortality. Surgeons have to concentrate on timing of surgery as the permission of treatment PFF depends on exceeding 24 h to surgery in 15% at the most [5]. Further there are international guidelines and recommendations for surgical treatment in detail, anaesthesia and postoperative physical therapy, which advises consistent approach [6].

The objective of our study was therefore to evaluate the mortality rate after surgical therapy in every patient with PFF and to analyze both, well-known and any other potential cofactors which might influence the mortality rate within 90 days after surgery in PFF.

## Methods

**Fig. 1:**
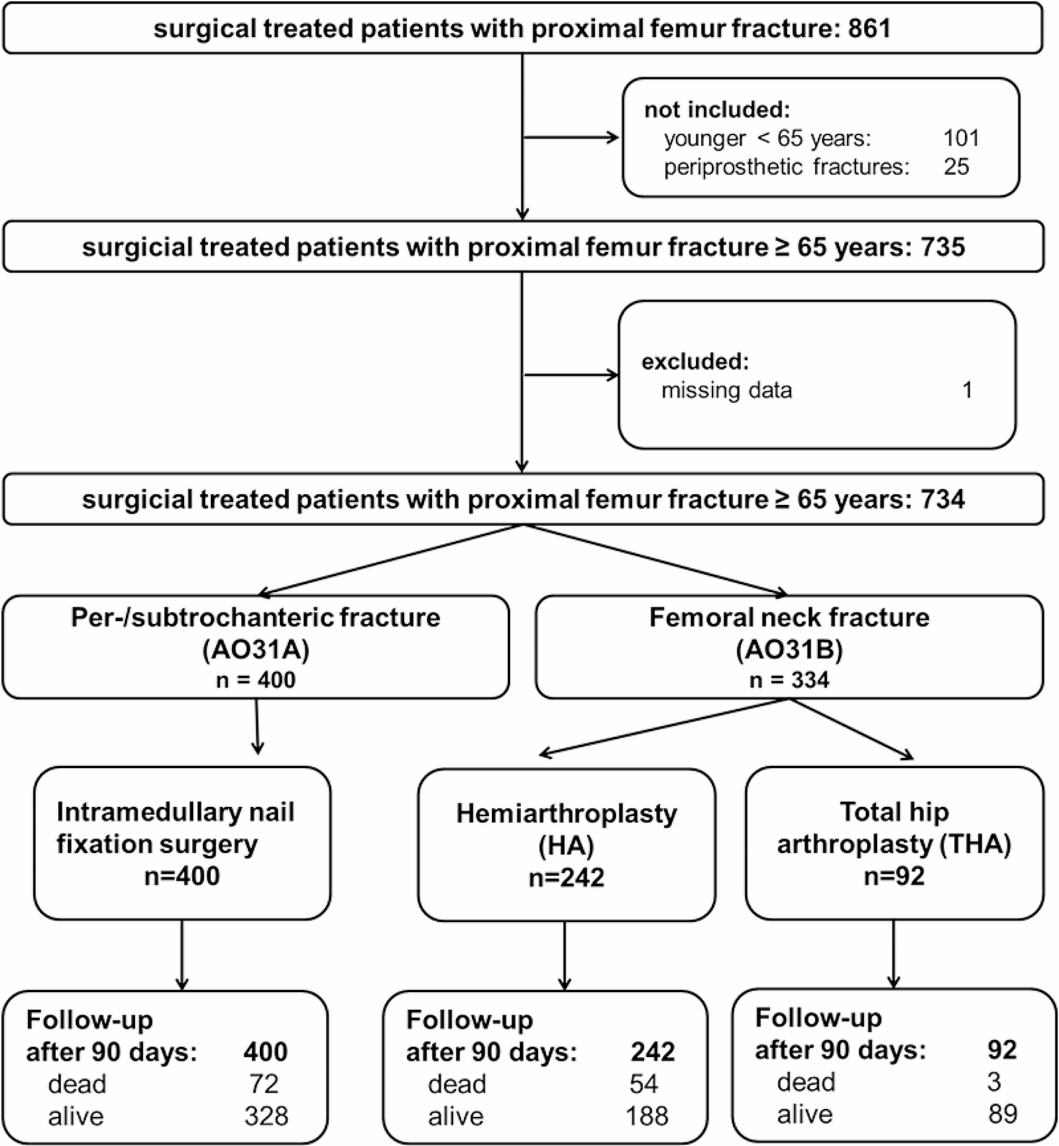
Flowchart

This retrospective cohort study identified all geriatric patients with a PFF who were treated by multiple surgeons at a single academic level 1 trauma center between 01/2019 and 12/2020. Patients aging 65 years or older were included with a PFF (femoral neck, pertrochanteric and subtrochanteric fractures, Fig. 1). Exclusion criteria were isolated greater trochanteric fractures (type AO31A1.1) without surgical treatment, periprosthetic fractures or revision surgery in previous fixed fractures. Patients with other concomitant injuries were not excluded, but patients with previous fractures, surgeries of the proximal femur or periprosthetic fractures were exluded. All patients received a geriatric screening in the emergency department called “Geriatrie-Check” to identify them as geriatric patients [7].

Demographic data such age, gender, BMI, comorbidities including the Charlson Comorbidity Index CCI [8] and ASA American Society of Anaesthesiologists classification [9], fracture morphology and medication were collected. Concomitant injuries and their treatment, duration to surgery and length of stay were recorded.

The performed surgical procedure depends on the fracture morphology. Femoral neck fractures (type AO31B) were treated by a total hip arthroplasty (THA) or hemiarthroplasty (HA) due to patients’ preoperative mobility and comorbidities. In pertrochanteric fractures (type AO31A) intramedullary nailing with or without femoral neck cement augmentation was performed, in subtrochanteric fractures with an additional cable cerclage after minimal-invasive open reduction of the fracture. In some cases of type AO31A fractures due to comminuted fracture full weight bearing was not allowed and limitation on 20 kp for six weeks was recommended.

The surgery was performed in spinal, general or regional anesthesia. The method depends on comorbidities and previous taken blood thinners and should be as mild as possible.

We recorded complication rate and divided surgical and non-surgical complications respectively our local complication registration.

The mortality up to 90 days was registered by the hospital information system, a postal or telephone request with the patients or their relatives. We had completed data about mortality, but not about revision rates, depending on the following admission to other hospitals in any reasons.

We divided all patients into a survival group with patients who were alive after 90 days and a death group with patients who were dead at 90 days after their surgery. We choose the 90 day time interval as at least time for recovery and therefore possible direct influence on mortality and as the follow-up time in our local registry.

### Statistical analysis

Data description was based on medians, interquartile ranges (IQR) and ranges for continuous values and absolute and relative frequencies for categorical values. Differences between groups were analyzed using Mann Whitney U test for not normally distributed continuous values and chi-square test for categorical values. A multivariable logistic regression model was used to identify factors associated with mortality after 90 days. In the final model only factors with a significance level less than 0.2 in the univariate analysis were included. Odds Ratio (OR) with 95% CIs were also estimated. Cumulative incidences for death up to 90 days of the patient were calculated with the Kaplan-Meier estimator.

A *p*-value threshold of 0.05 results was considered statistically significant. All data analyses were carried out using SPSS (release 28.0 for Windows).

An ethics approval for this study was obtained from the Ethics Committee of the University Medicine Carl Gustav Carus, TU Dresden in 2021 (BO-EK 189,032,021).

## Results

From 01/2019 until 12/2020 in total 734 patients with PFF fulfilled the inclusion criteria. The proportion of female patients was 68% (*n* = 500) and median age 85 years (65.2–101 years). 84% (*n* = 613) were identified as geriatric patients by the geriatric screening [7] in the emergency department and 78% (573 patients) had ASA III or IV and median CCI was 3 (range 0–12). The characteristic of the study population is summarized in Table 1.

**Table 1:** Baseline characteristics of 734 included patients

	Overall (*n* = 734)		Alive at 90 days (*n* = 605)		Dead at 90 days (*n* = 129)		*p*-value
Median age, yrs (IQR)	85.1	(10.1)	84.3	(9.5)	89.7	(8.7)	**< 0.001‡**
Female, *n* (%)	500	(68.1)	418	(69.1)	82	(63.6)	0.222†
Median BMI, kg/m^2^ (IQR)	23.9	(5.2)	24.0	(5.0)	23.3	(5.1)	0.003‡
**Comorbidities, ** *n * **(%)**							
Dementia	254	(34.6)	182	(30.1)	72	(55.8)	**< 0.001†**
Chronic renal insufficiency	360	(49.0)	275	(45.5)	85	(65.9)	**< 0.001†**
Diabetes	188	(25.6)	150	(24.8)	38	(29.5)	0.271†
Cerebrovaskular disease	174	(23.7)	137	(22.6)	37	(28.7)	0.143†
Chronic pulmonary disease	101	(13.8)	78	(12.9)	23	(17.8)	0.139†
Myocardial infarction	52	(7.1)	38	(6.3)	14	(10.9)	0.066†
**Antithrombotics, ** *n * **(%)**							
No Antithrombotics	533	(72.6)	455	(75.2)	150	(60.5)	**0.001†**
Vitamin K antagonist	20	(2.7)	16	(2.6)	4	(3.1)	0.751†
DOAC	162	(22.1)	121	(20.0)	41	(31.8)	**0.002†**
Thrombocyte aggregation inhibitor	8	(1.1)	5	(0.8)	3	(2.3)	0.130†
**ASA grade, ** *n * **(%)**							
1 to 2	161	(21.9)	154	(25.5)	7	(5.4)	**< 0.000†**
3 o 4	573	(78.1)	451	(74.5)	122	(94.6)	
**Median CCI (IQR)**	3	(3)	3	(3)	4	(3)	**< 0.001‡**
Median preop. Hb level, mmol/l (IQR)	7.7	(1.4)	7.8	(1.3)	7.3	(1.7)	**0.001‡**
Median preop. GFR, ml/min/1.73 m^2^ (IQR)	60.0	(36.0)	62.5	(33.5)	45.0	(34.0)	**< 0.001‡**
Median preop. INR (IQR)	1.1	(0.2)	1.1	(0.2)	1.2	(0.3)	**< 0.001‡**

†Chi-squared test; ‡Mann-Whitney U test

ASA, American Society of Anesthesiologists; BMI, Body Mass Index; CCI, Charlson Comorbidity Index; DOAC, Direct Oral Anticoagulants; GFR, glomerular filtration rate; Hb, haemoglobin; INR, International Normalized Ratio; SD, standard deviation

Overall, 400 patients (55%) received cephallomedullary nail fixation, 242 patients (33%) received HA and 90 patients (13%) THA (see Table 2).

**Table 2:** Perioperative variables of 734 included patients

	Overall (*n* = 734)		Alive at 90 days (*n* = 605)		Dead at 90 days (*n* = 129)		*p*-value
**Surgical procedure, ** *n* ** (%)**							
Hemiarthroplasty	242	(33.0)	188	(31.1)	54	(41.9)	**< 0.001†**
Total Hip Arthroplasty	92	(12.5)	89	(14.7)	3	(2.3)	
Intramedullary nail fixation	400	(54.8)	328	(54.2)	72	(55.8)	
**Anesthesia, ** *n * **(%)**							
Spinal	379	(51.6)	320	(52.9)	59	(45.7)	0.328†
General	312	(42.5)	251	(41.5)	61	(47.3)	
Regional	43	(5.8)	34	(5.6)	9	(7.0)	
**Median time to surgery, h (IQR)**	22.2	(17.8)	22.0	(18.7)	23.2	(17.3)	**0.156‡**
< 24h, *n* (%)	433	(59)	363	(60.0)	70	(54.3)	0.268†
24-48h, n (%)	246	(33.5)	195	(32.7)	51	(39.5)	
> 48h, *n* (%)	55	(7.5)	47	(7.8)	8	(6.2)	
Median duration of surgery, min (IQR)	65.0	(41.0)	65.0	(43.0)	62.0	(35.0)	0.945‡
Median duration of anesthesia, min (IQR)	146.0	(57.0)	145.0	(55.0)	155.0	(60.0)	**0.007‡**
Median nights at ICU (IQR)	0.0	(1.0)	0.0	(1.0)	1.0	(2.0)	**< 0.001‡**
Median Hb 3 d postop., mmol/l (IQR)	5.7	(1.1)	5.7	(1.1)	5.6	(1.1)	0.274‡
Median blood loss, I (IQR)	1.3	(0.9)	1.2	(0.8)	1.5	(1.2)	**0.003‡**
Median EC total, number (IQR)	0.0	(2.0)	0.0	(2.0)	1.0	(2.0)	**< 0.001‡**
**mobilization postop., ** *n * **(%)**							
No mobilization	40	(5.4)	8	(1.3)	32	(24.8)	**< 0.001†**
Partial weight-bearing	68	(9.3)	59	(9.8)	9	(7.0)	
Full weight-bearing	626	(85.3)	538	(88.9)	88	(68.2)	

†Chi-squared test; ‡Mann-Whitney U test

EC, erythrocyte concentrates; Hb, haemoglobin

Median duration time to surgery was 22.2 h (range 0.5 h – 33.35 days). 433 patients (59%) received their surgery during 24 h, 246 patients (34%) between 24 and 48 h. 55 patients (8%) had a delayed surgery after 48 h. The majority with 402 patients (55%) had their surgery during the day time from 8.00 to 15.30, 271 patients (37%) from 15.31 to 24.00 and 61 patients (8%) from 0.01 to 8.00 o`clock. There was no significant difference between the mortality after 90 days in these three groups (*p* = 0.895).

The length of stay postoperatively on the intensive care unit (ICU) varied from 0 to 32 days (median 0 days). 132 patients (18%) were there for one night, 88 patients (12%) for two to seven nights and 19 patients (3%) for more than seven nights. The highest prevalence of 90d-mortality was seen in the group with the longest ICU stays (63%, *p* < 0.001).

The most frequent concomitant injuries were head injuries in 69 patients (9%), mostly craniocerebral trauma and in 7 patients (1%) with intracranial bleeding but no one with surgical intervention. The second frequent injury were wrist fractures in 51 patients (7%), of which 23 (3%) needed surgical treatment.

145 patients (20%) suffered from any adverse events. While just 21 patients (3%) had a surgical complication, from which 9 patients (1%) needed a revision surgery, the major proportion, 132 patients (18%) suffered from non-surgical complications, for instance pneumonia, which is caused by less mobilization before or after surgery.

Surgical complications are summarized in Table 3.

**Table 3:** adverse events (AE)

	Overall (*n* = 734)		Alive at 90 days (*n* = 605)		Dead at 90 days (*n* = 129)		*p*-value
**Adverse events any, ** *n * **(%)**	145	(19.8)	92	(15.2)	53	(41.1)	**< 0.001†**
Surgical	21	(2.9)	13	(2.1)	8	(6.2)	**0.012†**
Non-surgical	132	(18.0)	83	(13.7)	49	(38.0)	**< 0.001†**
Revision surgery	9	(1.2)	4	(0.7)	5	(3.9)	**0.003†**
**complications**							
Decubitus	8	(1.1)	2	(0.3)	6	(4.7)	**< 0.001†**
Non-surgical diseases	82	(11.2)	47	(7.8)	35	(27.1)	**< 0.001†**
Dislocation	1	(0.1)	1	(0.2)	0	(0.0)	0.644**†**
Thrombembolic events	11	(1.5)	8	(1.3)	3	(2.3)	0.395**†**
Cognitive impairment	24	(3.3)	14	(2.3)	10	(7.8)	**0.002†**
Nervous lesion	4	(0.5)	3	(0.5)	1	(0.8)	0.696**†**
Fracture	1	(0.1)	1	(0.2)	0	(0.0)	0.644**†**
Impairment of wound healing	1	(0.1)	0	(0.0)	1	(0.8)	**0.030†**
Postoperative hematoma	4	(0.5)	2	(0.3)	2	(1.6)	0.088**†**
Periprosthetic / periimplant infection	5	(0.7)	2	(0.3)	3	(2.3)	**0.012†**
Other	33	(4.5)	20	(3.3)	13	(10.1)	**0.001†**

†Chi-squared test

129 patients died within 90 days, the mortality rate to 90 days after surgery was 18%, whereas 31 patients (4%) died during the hospital stay, occasionally according to their decision in their patient’s provision.

We performed logistic regression to identify factors associated with mortality after 90 days in patients with PFF after surgery (see Table 4). No significant association was found between modifiable surgical parameters as time to surgery, type and time of surgery nor time or type of anesthesia.

**Table 4:** Results of logistic regression models – factors associated with 90d-mortality undergoing surgery in PFF

Variable	Ref.	OR	95% CI	*p*-Value
Age (yrs)	per 1 year	1.080	(1.034; 1.128)	> 0.001
BMI (kg/m²)	per 1 kg/m²	0.915	(0.857; 0.978)	0.009
Comorbidities				
Dementia	No	2.805	(1.616; 4.869)	> 0.001
Chronic renal insufficiency	No	0.765	(0.401; 1.459)	0.416
Cerebrovaskular disease	No	1.303	(0.714; 2.379)	0.389
Chronic pulmonary disease	No	0.965	(0.468; 1.992)	0.924
Myocardial infarction	No	0.824	(0.319; 2.125)	0.689
Antithrombotics				
DOAC	No	1.235	(0.682; 2.238)	0.486
Thrombocyte aggregation inhibitor	No	2.295	(0.344; 15.319)	0.391
ASA grade 1/2	3/4	1.199	(0.487; 2.952)	0.694
Charlson Comorbidity Index	per 1	1.170	(1.018; 1.345)	0.027
preop. Hb level (mmol/l)	per 1 mmol/l	1.009	(0.777; 1.310)	0.948
preop. GFR (ml/min/1.73 m²)	per 1 ml/min/1.73 m²	0.984	(0.969; 0.999)	0.041
preop. INR	per 1	1.410	(0.832; 2.389)	0.202
Surgical parameters				
THA	HA	1.836	(0.420; 8.020)	0.419
THA	PFN	2.074	(0.462; 9.308)	0.341
blood loss (I)	per 1 l	1.062	(0.758; 1.489)	0.726
EC total (number)	per 1 EC	0.978	(0.828; 1.156)	0.794
Anesthesia				
General	Spinal	0.776	(0.435; 1.386)	0.392
General	Regional	0.519	(0.148; 1.815)	0.305
duration of anesthesia (min)	per min	1.005	(0.999; 1.011)	0.112
nights at ICU	per night	1.117	(1.025; 1.218)	0.012
adverse events				
non-surgical	No	2.276	(1.269; 4.083)	0.006
surgical	No	3.226	(0.854; 12.193)	0.084
mobilization				
full weight-bearing	partial	0.748	(0.274; 2.04)	0.570
Full weight-bearing	None	10.493	(3.612; 30.479)	> 0.001

**Fig. 2:**
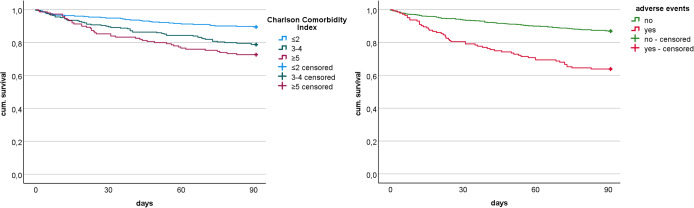
Kaplan-Meier-curve (a Charlson Comorbidity Index, b adverse events)

ASA, American Society of Anesthesiologists; BMI, Body Mass Index; CCI, Charlson Comorbidity Index; DOAC, Direct Oral Anticoagulants;

GFR, glomerular filtration rate; EC, erythrocyte concentrates; Hb, haemoglobin; INR, International Normalized Ratio;

Significant association with mortality after 90 days were only found for age, BMI, preoperative existing dementia, higher CCI, low preoperative GFR, nights at ICU, postoperative non-surgical adverse events and impossible mobilization, see Fig. 2.

## Discussion

PFF often result from low-energy trauma, as a typical osteoporosis-related fracture in female patients. PFF can have serious consequences for the patients, including mobility limitations, higher long-term disability and care and increased mortality rates. The mortality rate after PFF can be an important consideration for patients and surgeons when determining treatment options and assessing the overall outcome. We recorded every life status after 90 days and found a mortality rate about 18% and 4% in-house mortality after PFF. In the literature, mortality rates at 90 days after PFF ranges from 9 to 22% depending on several modifiable factors like preoperative optimisation, reducing time to surgery, kind of anaesthesia [10–19] or patient-related unmodifiable factors like patients age, sex, comorbidities, kind of fracture or concomitant injuries as well as frailty [20].

Modifiable perioperative factors to reduce mortality and improve patients’ outcomes getting more interesting in the last decades. International guidelines recommend early surgery [21–27], whereas the definition of early surgery ranges from six to 72 h. In our study 59% patients received their surgery during the first 24 h after hospital admission and 33.5% between 24 and 48 h. The postponement up to 48 h depends on the previous recommended time for the treatment of femoral neck fractures with a hip arthroplasty or in cases of eliminating antithrombotic drugs. The delay after 48 h was often required to preoperatively optimize critical comorbidity conditions, but when the patients resources allow and there is no specific reversible contraindication to early surgery, PFF should be surgically treated within 24 h [28]. The last years timing to surgery gets more important, particularly as the German government required surgery within 24 h for all patients with permission to treat in general. In our study with 41% of delayed surgery after 24 h we found no significant association between time to surgery and mortality rates. Two investigations about timing of surgery in PFF found no differences in mortality when optimizing patients preoperative conditions leads to postponement [29, 30]. No differences were seen in mortality in patients with medical indications for postpone after 24 h, but higher pressure scores and urinary tract infections when time to surgery increased [31]. An registry analysis reported about similar results. Patients under best possible conditions whether treated within 24 or 48 h had no significant differences regarding in-house mortality rate [32].

There are a lot of patient-related, but unmodifiable factors, which should be taken into consideration. Older age and pre-existing severe comorbidities such as cardiovascular or pulmonary disease or dementia may be at increased risk for mortality after PFF [33, 34]. We measured comorbidity with the CCI and the ASA Score, whereas the accuracy to assess mortality after 90 days and 1 year differs between these scores [35]. Our results confirm higher mortality rates in higher CCI and pre-existing dementia as well as a lower BMI and leads to the assumption to optimize patients before surgery to reduce adverse events, which are associated with higher mortality as well. Dementia is known as an independent risk factor for mortality after one year and the severity of dementia in hip fracture patients as a risk factor for mortality within 6 months and one year [36]. Patients should be screened therefore for delirium risk and the treatment should include prevention as well as early detection and therapy.

We found concomitant injuries in patients with PFF in every tenth for head injuries and every 14th distal radius fractures, whereas the incidence of these other injuries showed no significant association to the mortality rate. In a matched pair analysis about concomitant fractures in patients with PFF higher CCI in these patients and a longer hospital stay than patients with an isolated hip fracture but no difference in mortality rate were reported. The reason for the low in-house mortality rate about 2% were seen in an early mobilization program with full weight bearing after surgery for every patients regardless isolated hip fracture or concomitant fracture [37]. We can confirm these results as we found a significant higher mortality rate in patients without any mobilization due to their comorbidities and disability to walk before their fall. About nursing home inhabitants, suffering from severe osteoporosis, dementia and sarcopenia similar findings are published and improving the ambulant assessment and therapy of these complicating factors is recommended [38].

The choice of the anesthesia modality remains a controversial issue in the literature [39–42]. In our data, no significant association was found between type of anesthesia. The majority of the patients underwent spinal anesthesia, which is our preferred modality in geriatric patients.

It is well known that PFF can have a significant impact on patients’ quality of life, leading to decreased independence, increased healthcare costs, and a greater need for long-term care. These factors may contribute to increased mortality rates after PFF.

Our data reveal the non-surgical adverse events and poor preoperative general condition of the geriatric patients as risk factors for 90-day mortality. Therefore, the author advertise for a new understanding of PPF as an end of life disease with high 90-day mortality rate. For us the PFF fracture poses a symptom of decompensation of the general patient´s condition and often non-surgical, internal medicine disease underlining the need of interdisciplinary management to optimize the entire treatment of these vulnerable patients.

## Limitation

Main limitation is the retrospective analysis of a single-center study of two years. There were no information about the previous mobilization, which has an influence on the postoperative mobility grade. Furthermore we don’t have any information about following additional injuries or complications treated in other hospitals, which could influence mortality within the 90 days. In comparison to larger register studies, the strengths of this study are the complete follow-up data about mortality and detailed information about the important patient- and treatment-related cofactors.

## Conclusion

The proportion of patients who died until 90 days after surgery is still high. It is less extend influenced by surgical and anaesthesiologic factors than by patient-related factors like age or lower BMI. Physicians should be aware of the importance of avoiding adverse events and the importance of patients mobilization to reduce mortality and improve patients’ outcome.

## Data Availability

The datasets generated during and analyzed during the current study are available from the corresponding author on reasonable request.
